# Crime, Disorder, and Territorial Stigmatization: Older Adults Living in Deprived Neighborhoods

**DOI:** 10.1093/geront/gnac159

**Published:** 2022-10-19

**Authors:** Afsaneh Taei, Håkan Jönson, Marianne Granbom

**Affiliations:** Department of Health Sciences, Lund University, Lund, Sweden; School of Social Work, Lund University, Lund, Sweden; Department of Health Sciences, Lund University, Lund, Sweden

**Keywords:** Deprived area, Hirschman’s theory, Neighborhood reputation

## Abstract

**Background and Objectives:**

The existence of social problems, crime, and a diminishing sense of community are acknowledged challenges to residents of deprived neighborhoods. In research on deprived neighborhoods in Sweden, the perspectives of young residents and adults of working age dominate. This study explores how older adults in deprived neighborhoods in Sweden experience crime and disorder, and how they adapt and respond to these problems and the neighborhood’s poor reputation.

**Research Design and Methods:**

Semistructured interviews were conducted with 22 older adults who had lived 5 years or more in deprived areas of two cities in Sweden. Data were analyzed using Hirschman’s theory of exit, voice, and loyalty with a thematic analysis.

**Results:**

Most residents had positive things to say about their homes and neighborhoods, even if criminal acts such as shootings, drug dealing, arson, burglary, and knife attacks were part of everyday life. The residents attempted to manage these events with various strategies. Exit strategies included relocation and forms of adaptation and detachment. They used several voice strategies to actively try to solve the problems and engage with the community. Loyalty strategies—and relativizing—were used to defend their neighborhood reputation.

**Discussion and Implications:**

The findings show we should move on from generalized notions of older adults as passive victims of their environment and highlight that some older adults are active agents in building communities in deprived neighborhoods. City improvement programs should extend support to older adults who wish to engage. Approaches are identified which may strengthen older adults’ contributions in such neighborhoods.

## Background

There are socially deprived neighborhoods in many cities. In Europe, they are commonly characterized by a high level of unemployment and poverty, changes in ethnic composition, street-crime, and social unrest. A diminishing sense of community, social problems, and the tainted character of these areas convince some residents to move out, and many say they would live elsewhere if they could do so (Buffel et al., 2013; Permentier et al., 2011; Van der Land & Doff, 2010). Some residents avoid public spaces out of fear or feel excluded from shops and local activities dominated by cultures that they are not accustomed to, thereby decreasing their social activities (Van der Land & Doff, 2010).

This article addresses the experiences, views, and strategies of older people who live in socially deprived neighborhoods in two cities in southern Sweden (the terms neighborhood and area will be used interchangeably in this article to reflect the similarity in use between the corresponding Swedish terms “grannskap” and “område”). In general, deprived neighborhoods in Sweden have many characteristics described in the international literature, but the housing standard is good, and public housing companies (allmännyttan) are by far the largest providers of rental housing. The neighborhoods are designated “deprived areas” by the Swedish police in a national list updated annually (Polisen, 2022): what constitutes a deprived area, besides socioeconomic factors, are a high population turn over, high levels of crime, drug dealing in public spaces, the closure of local services, and a reluctance to report the crime, harassment of victims and witnesses. Outburst of social unrest occur, where cars are set on fire, rocks thrown at buses, and the ambulance and fire brigade may have to operate with police escort. The Swedish police have been criticized for the stigmatizing effects of publishing the list of deprived areas (Larsen & Delica, 2019).

To date, few studies of socially deprived areas have focused on older people as active agents in their neighborhoods. Some researchers concentrate on how life in these neighborhoods affects older people’s health and well-being negatively (Beard et al., 2009; Choi & Matz-Costa, 2018; Julien et al., 2012; Shepperd et al., 2022; Wu et al., 2015), compounded by restricted outdoor mobility and possibilities for participation (Annear et al., 2014; Danielewicz et al., 2017; Rosso et al., 2011). The situation of older people in deprived neighborhoods has also been investigated as a form of social exclusion (Buffel et al., 2013; Scharf et al., 2005), and several studies have focused on the factors that encourage older people to relocate to other areas or to consider doing so (Hillcoat-Nallétamby & Ogg, 2014; Permentier et al., 2011). Comparisons between “now and then,” like “us and them,” have been prominent in interviews with older residents. A previous community or safety in the neighborhood—sometimes described in idyllic terms—has been lost (Buffel et al., 2013; Dahlberg, 2020; Van der Land & Doff, 2010). When new populations move in, older people who have lived there for a long time may feel they are “isolated survivors of a previous generation,” threatening their feelings of “togetherness” (Victor et al., 2009, p. 207). This focus is also present in attempts to theorize the reputational dynamics of deprived areas. Wacquant et al. (2014) propose a typology of strategies which residents may use to handle territorial stigma: submission strategies which include attempts to exit or withdraw; recalcitrant/resistance strategies may be expressed as loyalty or pride in the neighborhood; criticism of externally produced labels; or activities that embrace the community and defy the stigma (Wacquant, 2008; Wacquant et al., 2014). Wacquant et al. (2014) identifies young sons of migrant tenants as the type likely to resist denigratory images among outsiders and to express pride. In contrast, “a longtime elderly homeowner” (Wacquant et al., 2014, p. 1276) with relatively secure finances and family members living nearby is the type likely to stress differences in relation to the neighborhood and to retreat into the family ambit. Although these examples were primarily used to illustrate positions in the typology, the latter align with a tendency to focus on ways that older people are affected by crime, unrest, and the poor reputation of the area. Studies of active responses usually focus on younger populations, as is the case in Sweden (Johansson & Olofsson 2011; Lalander & Sernhede 2011).

While it is important to highlight these aspects, there is an associated risk that the research focus reproduces established stereotypes of old age as a period of vulnerability, pastness, and withdrawal. Thus far, little attention has been paid to the ways older people act on social problems, contribute to social life in deprived areas or defend the reputation of their neighborhoods. A study by Wanka (2018) has highlighted activities among older residents that contradict the idea that older people always exit or withdraw, but the matter has not been investigated in depth.

The aim of this study was to explore how older adults who live in deprived neighborhoods in Sweden experience crime and disorder in everyday life. What strategies do they use to manage and adjust to events? How do they reason about their neighborhood’s poor reputation? Is it possible to identify approaches which, with development and support, could further strengthen the older people’s contribution to these neighborhoods?

In order to analyze these positions and responses, a conceptual framework originally developed by Hirschman (1970) will be used. According to Hirschman, people respond to problematic conditions in their immediate environment by using “exit, voice, or loyalty” (EVL) strategies. This framework can be applied when identifying and classifying older adults’ strategies when confronted with crime and disorder (Permentier et al., 2007, 2011; Van der Land & Doff, 2010). To exit is to move away from the deprived neighborhood, although, as Van der Land and Doff (2010) note, various forms of avoidance, withdrawal, and isolation can also constitute exit strategies. The concept of voice spans many of their attempts to deal with problems, ranging from informal discussions among neighbors and direct action when facing problems to collective action and formal organizations. Loyalty affects people’s tendency to use exit or voice, and neighborhood attachment has been used to operationalize the concept (Permentier et al., 2011). Loyalty is typically associated with voice strategies; residents that express pride and embrace the community of their neighborhoods are more likely to act upon perceived problems (Van der Land & Doff, 2010). Neglect is sometimes included as an additional response: the opposite of loyalty, or part of a loyalty scale, it appears in reasoning about the type of distancing, detachment, and withdrawal associated with exit responses (Permentier et al., 2007). Although generally considered behavioral, the EVL framework comprises cognitive and emotional components, and the present analysis is based on descriptions of behaviors as well as perceptions and feelings relating to crime, disorder, and the character of the area.

## Methods

### Study Context

The current study is part of the project Older Adults Living in Disadvantaged Neighborhoods: A Mixed-Methods Study of Homes, Neighborhood Transitions, and Wellbeing. It is a 4-year project that uses qualitative interviews, photo-elicitation data (*n* = 39), and quantitative survey data (*n* = 360) to investigate older adults’ identity, participation, and well-being in deprived neighborhoods in Sweden, both urban and rural (Granbom et al., 2022). The present study uses qualitative interview data from 22 older adults who live in deprived urban areas in southern Sweden.

The study targeted older adults aged 65 and older, who had lived for at least 5 years in any of the deprived areas, listed by the Swedish police, in the coastal cities that we will refer to as Springfield and Franklin. Springfield has 110,000 inhabitants and four deprived areas, according to the police, one of which is downtown, and the other three are city districts, each with a community center. Most housing is publicly owned rental apartments. Franklin has 46,000 inhabitants and one deprived area. It is not downtown, and because the community square and its stores were repeatedly vandalized, it was replaced with more housing a few years ago. Most housing is rental apartments owned by private companies. In Springfield, participants were recruited through the older adult center, the public library, and nonprofit organizations that promote integration and community. After recruiting twelve participants, we had to change our strategy because of the coronavirus disease 2019 (COVID-19) pandemic restrictions. For Franklin, we thus ordered the addresses for all adults over the age of 65 in the area in question from SPAR (the Swedish state personal address register), wrote with information to 20 randomly chosen residents, and followed up with a phone call. This was done in four stages and resulted in a further 10 participants. Recruitment took place between September 2020 and April 2021. In all, the study comprised 22 participants (see Table 1), of whom six were men and 16 women, with a mean age of 74 ranging from 65 to 92 years; two were married and did the interviews together; and three were born in Iran and were interviewed in Persian, the others being interviewed in Swedish.

**Table 1.** Respondents (*N* = 22)

Participant characteristics	*n* (%)	*M* (*SD*)
Gender		
Male	6 (27)	
Female	16 (73)	
Age		74 (8)
Place of residence		
Springfield	12 (55)	
Franklin	10 (45)	
Lives alone		
Yes	16 (73)	
No	6 (27)	
Years as resident		24 (18)
Type of housing		
Rented	19 (86)	
Owned	3 (17)	
Level of education		
Nine years or less	8 (36)	
Ten to 12 years (high school)	11 (50)	
Over 12 years (university)	3 (14)	
Born in Sweden		
Yes	16 (73)	
No	6 (27)	

*Note*: *SD* = standard deviation.

### Data Collection

We developed an interview guide with topics such as residents’ perception of the character and reputation of the neighborhood, everyday life in the neighborhood, relationship with the neighbors, social participation, changes in the neighborhood over time, identity and belonging, thoughts about their future housing situation and the future of the neighborhood. The interview guide was tested with three respondents. As only minor adjustments to the interview guide were needed after that, we included those three respondents in the final sample. The COVID-19 pandemic meant the majority of interviews could not be done face to face at home, and instead were conducted using video conferencing software or by phone, at a senior citizen center, or outdoors close to home. All interviews were digitally recorded and transcribed verbatim.

### Data Analysis

**Figure 1.**
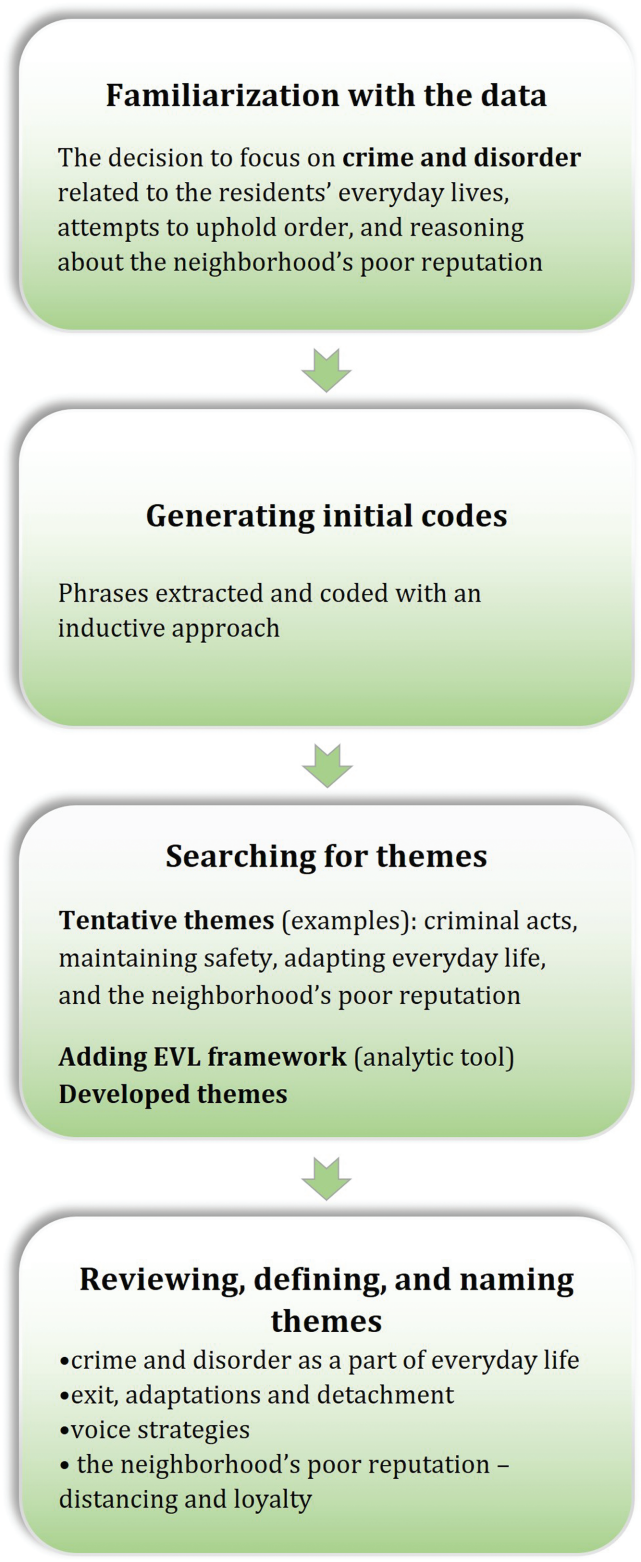
Overview of the thematic data analysis process (adapted from Braun & Clarke, 2012). For more detail, see Supplementary Material. EVL = exit, voice, or loyalty.

The study used a thematic analysis (Braun & Clarke 2012). We used an inductive approach at first, initially when the authors met during data collection and afterward when familiarizing ourselves with the data. During that phase, the subject of crime and disorder emerged—related to the residents’ everyday lives, attempts to uphold order, and reasoning about the neighborhood reputation—and we decided to concentrate on this specific subject. The phrases with bearing on the subject were extracted and coded by the first author, and tentative themes such as criminal acts, maintaining safety, adapting everyday life, neighborhood reputation, and participants’ contributions and strategies were developed. The coding and preliminary themes were repeatedly discussed at meetings of all the authors to improve the trustworthiness. The data revealed, at first, puzzling contrasts. Participants described horrible events and dangerous acts that they had encountered or witnessed. The responses or strategies to those events were, however, drastically different among the participants. Some expressed avoidance. Others surprised us by describing how they interfered in drug dealing attempts. To better understand these contrasting quotes, and complex patterns of strategies used by the respondents to deal with crime, disorder, and neighborhood reputation, we, as the next step, implemented the EVL framework (Hirschman, 1970; Permentier, 2007) as an analytical tool. We related descriptions of crime and responses to EVL strategies. We also compared how loyalty to the neighborhood influenced both exit and voice strategies. We found exit strategies to be used both as a way to continue daily life as well as they appeared as outcomes of people detaching themselves from the neighborhood. The finalized results include the themes Crime and Disorder as a Part of Everyday Life; Exit, Adaptations, and Detachment; Voice Strategies; and The poor reputation of the neighborhood—Distancing and Loyalty. For more details on the analytic process, see Figure 1 and Supplementary Material. The analysis was facilitated by NVivo software.

### Ethical Considerations

All respondents gave written consent before the interview. Names of individuals and cities were anonymized. The Swedish Ethical Review Authority approved the study (Reg. No. 2020-03468).

## Results

### Crime and Disorder as Part of Everyday Life

All respondents had experienced criminal acts and disturbances in their everyday lives. This ranged from shootings, knife attacks, drug dealing, burglary, arson, and property damage to disturbances such as littering, disorderly youths, drunk homeless people breaking into basements to sleep, kids playing loudly at night, cars driving too fast and honking horns, and a lack of respect for apartment rules. Of all the crimes they perceived as a problem, drug dealing was both the most common and particularly disturbing, as Karin (71, Springfield) put it: “I see them selling drugs right under my balcony in the middle of the day.” Drug dealers used wastepaper baskets and bushes to store drugs or as pickups, resulting in the frequent presence of the police—“The police are here all the time”—which was characteristic of the area.

Arson was the other criminal act noted by the respondents. In Franklin, in particular, it had a significant impact on residents and the neighborhood. A couple of months before he was interviewed, Anders (74, Franklin) and his wife had had to leave their apartment in a seven-story building one night because “someone set fire to the attic,” and after being evacuated to a hotel, their landlord provided them with another apartment in the same area. The local shopping center in Franklin was repeatedly set on fire over the course of several years and was eventually replaced by housing cooperatives. The lack of access to a supermarket, bakery, hairdresser, restaurants, ATMs, and post office left residents disappointed and frustrated. “If you need to post a letter, you have to take the bus downtown!” Barbro (75, Franklin) stated. The absence of a local shopping center limited the possibilities of social interaction that affected the sense of community, because “it was like the center, the heart of the neighborhood” as Monika (67, Franklin), said. Other fire incidents involved basement storage and recycling rooms, motor vehicles, and benches in the local parks.

### Exit, Adaptation, and Detachment

As a response to crime and disorder in the area, some residents expressed the wish to move away. Karin (71, Springfield), talking about drug dealing and the constant police presence, finished by saying, “I don’t want to stay, it’s as simple as that.” Laleh (66, Springfield) had never felt attached to the area and wanted to move out. She agreed with outsiders that she lived in a bad neighborhood, but she could not afford to move elsewhere:

What they say is exactly how I feel. I know this is not a good area, especially when you hear it from someone else, then I feel … Sorry for this word in Persian! [laughter] … how helpless I am! To be forced to live here [laughter]! You know, I really have to, because the rent here is lower than in any other area.

Laleh was straightforward about her dislike for her neighborhood and wished she could afford to live somewhere with a significantly higher socioeconomic status where she felt she belonged, based on her life back in Iran. A sense of not belonging to the neighborhood, her difficulties coping with changes in her life since moving to Sweden, her lack of Swedish: all of it left her dissatisfied with her life situation and desperate to exit the neighborhood. Fatemeh (77, Springfield) stated: “I don’t think that just one person like me would want to do anything to make the neighborhood better; this neighborhood cannot be any better because it is both crowded and full of foreign residents.” She explained that as a person from Iran it was hard for her to accept and cope with the cultural differences she felt toward neighbors from Arabic countries. She believed that the neighbors’ family structures and habits were the main reason for the unclean, crowded character of the neighborhood, which would not change soon. Other types of exit strategies described in the literature (Van der Land & Doff, 2010) were evident in our interviews too, in comments about withdrawal and detachment from the neighborhoods’ problems. Exit strategies included avoiding being outdoors at night, avoiding certain areas, or choosing not to interact with other residents in the building. Given that those residents who withdraw from public spaces may be reluctant to participate in interviews about their neighborhood, it is likely that some details and subthemes of exit strategies and detachment were not captured in our study.

Alongside stories on exit and self-limiting measures, some respondents said they were not affected; they coped with the situation by distancing themselves from crime and disorder or by simply ignoring it. This type of detachment from the neighborhood was described by Kerstin (75, Springfield), who said she felt safe out in her neighborhood after dark. She knew about the drug dealing but her response was, “Well heck, if they’re doing that … That’s between the gangs, it has nothing to do with me.” She mentioned an incident when a young man was stabbed outside her building, and she rang the hospital to ask how to hold the compress to staunch the bleeding while waiting for the ambulance. Asked by the interviewer if she had not been afraid, she answered, “No, why should I be? I wasn’t the one threatened with a knife, that was others.” Her point was the violence did not pose a threat to her; her role was to take care of a victim.

In some cases, respondents cited past experience and their personality as the reason why they were not afraid, or had to take measures to avoid risk, or why they could distance themselves from the neighborhood’s problems. Birgitta (69, Springfield), for example, used to work late hours as a cleaner, which she said was a reason for her present reaction when encountering homeless people in the basement: “I’m confident as a person so I don’t get scared.” Similar responses were put down to the experience of having lived in more violent places around the world. Mina (74, Springfield), a migrant from Iran, said the neighborhood problems were negligible compared to what she had gone through in her home country.

What these examples show is that existing comparisons between now and then among older people in deprived neighborhoods did not necessarily focus on the deterioration of the area, and detachment was not always associated with exit strategies.

### Voice Strategies

Against this, attempts to uphold order and improve the neighborhood could be identified. Some respondents engaged in civic activities to improve the community by contributing to neighborhood safety or cleanliness. They picked up trash on their daily walks or removed stickers thought to be used as signals by drug dealers. They contacted landlords, local politicians, or municipality representatives to raise issues about safety in parks, lack of services, or traffic hazards. Some initiatives were private; others were at the instigation of the Swedish Union of Tenants, where many were active members. Of particular interest were respondents’ descriptions of voice strategies designed to stop crime and disorder, the three types being having a backup, building on relations, and using clever situational approaches.

The strategy of having backup appeared in three versions, the most obvious being to call the police or to threaten to do so. The second type of formalized backup was the neighborhood watch in each area. Sven (65, Springfield) said adults being out and about—and visible—at night made the drug dealers so uncomfortable they would avoid the area. What Sven described was a type of turf war between residents and dealers over open drug dealing. There was a localized, informal version outside the building where some respondents lived in Springfield, where a group of residents used backup strategies to create a context of intervention. Birgitta (69, Springfield) a neighbor and friend of Sven’s, described how she tried to disrupt the drug dealing by talking to the dealers directly, but evaluated the situation in advance and created a context of visibility that made her feel safe:

I know they’ve totally got my back, because when I’ve gone out there and … When the youngsters are hanging out at the corner of the other building, when I saw … when I go out to talk to them, I know that Kalle and Peggy are out on their balcony and Bill is out on his, watching, and then there are others watching, so they know … Yes, the youngsters know I have backup. We see you and we know who you are! So don’t do any of that shit!

Drug dealing was an acknowledged reality in the neighborhoods, but what provoked several respondents was when dealers did not bother to conceal their activities. Their reaction was to confront drug dealers as a group, using a type of activity that created public exposure while ensuring there was backup if there were trouble.

Building on relations was a strategy based on respondents’ knowledge of the families of the youngsters who misbehaved or were at risk. Birgitta explained why it was safe to intervene:

I’m not afraid of … I don’t feel scared or unsafe about it. Most times I know who the youngsters are, because it’s families that live here. And I’ve spoken to the parents and told them it can’t go on like this, them dropping trash and being loud and disturbing the neighbors at night, that they’ll have to go somewhere else.

Being known as a constructive person in encounters with young people made it easier for residents to voice concerns. Anders (65, Franklin) had worked as a caretaker in the area before he retired. He had once encouraged a group of young boys to help pick up trash in the area, and in exchange he persuaded the landlord to set up the football goals the boys had asked for. He described himself as well-known, with a history of good relations with young people in the neighborhood. His status made it possible for him to intervene.

Several situational approaches were described, ranging from witty rhetoric to being physically present. Birgitta said she disapproved of youngsters spitting outside her house and jokingly, she used to tell them that spitting on the ground was a sign of having a bad sex life. The emotional version of this approach was described by Saga (79, Springfield), who said that as an older person, she is likely to be perceived as more of a mother when talking to young people: “I told one guy, ‘You shouldn’t be here, you are not that old yet, you are like a small boy’. He teared up and got on his bike and took off.” Several respondents stressed the importance of a positive, constructive approach to people, particularly important when people had different ethnicities. Göran (74, Franklin) suggested native Swedes and migrants in the area could easily come into conflict. He described himself as someone who greeted his neighbors in a friendly manner regardless of ethnicity, but he knew of other residents who had negative views and “if you’re the kind to show you hate foreigners then they’ll resent you” or worse. What these comments communicated was that older adults who knew their neighbors and who were open and positive to others in the neighborhood were less likely to be affected by crime and disorder and had better chances to intervene.

### The Poor Reputation of the Neighborhood—Distancing and Loyalty

Manifestations of distancing and detachment or loyalty were intertwined as respondents commented on the development of the neighborhoods in terms of their reputation. A common opinion was that some problems had increased because of the influx of migrants. Several respondents expressed a positive view of migrants and were part of efforts to support newly arrived refugees, but still thought they constituted a reputational problem. Efforts to maintain and improve buildings and open spaces were mentioned as affecting the neighborhoods’ reputation, although the picture differed between the two cities: the Springfield residents praised the efforts of the municipality and the public housing company in refurbishing public squares and creating recreational areas; the Franklin residents were more muted and feelings were mixed about the municipality having refurbished its housing stock, and even having built new houses for sale to improve the neighborhood’s poor reputation by attracting wealthier families, because respondents worried about facing affordability challenges when the refurbishment costs were passed on to tenants. Monica (67, Franklin) went further, fearing that housing built for sale rather than for rent was a way of “getting rid of affordable housing for people on low incomes” in her area. Referring to an ideological standpoint, Monica expressed loyalty to an area that provided affordable housing for members of the working class, and she included immigrants in this population. Several respondents from Franklin commented sarcastically on the municipality’s attempt to improve the neighborhood’s image by rezoning the district to include streets where people lived in houses rather than apartments.

The reputation of the two neighborhoods as “deprived” was well known to residents and acknowledged as a stigma that affected them and their fellow residents. It was generally thought well deserved or as a matter of outsiders failing to see the true character of the neighborhood. A large proportion of newly arrived migrants, unemployment, poverty, crime, and disorder were reasons mentioned when respondents said the poor reputation of the neighborhood mirrored reality for those who lived there. Jan (92, Franklin) said that although it was wrong to judge everyone for some people’s failings, more people of Swedish origin were needed in the neighborhood.

Different evaluations among those who resided in the area and outsiders were mentioned in several interviews, as part of attachment and loyalty declarations. Barbro (75, Franklin) expressed a sense of shame about revealing her home address to outsiders:

It’s kind of sad that people who do not live here have many opinions, like, it’s like all my … when I used to work, I almost didn’t dare tell anybody where I lived, because all the time there was that talk, when we had meeting with different authorities, “those socioeconomically resource-poor, deprived areas,” felt too difficult, just like you were stamped as …

However, she defended the neighborhood’s reputation by arguing that outsiders could not know about the “sense of community and warmth” because they never spent time in the area: “here we help each other, shop together, and take care of one another.” She acknowledged that crime and disorder were real problems, but she appreciated the neighborhood’s societal cohesion.

Kerstin (75, Springfield) admitted that she too used to hold a prejudiced view before moving into the area, but linked it to a need for a high-status address she no longer had. She appreciated the sense of community among the migrant population:

Foreigners live here, and it’s so nice in my view. Because when I’m going down the street or go into a store and ask something, for instance … lots of foreigners going shopping. And I ask, “What’s this vegetable?” or “What’s this fruit?” then … everybody helps, they all say how you should use it. … Swedes don’t do that. It’s really great!

Another way of defending the neighborhood’s reputation was to downplay or relativize the crime and disorder. This was at its most prominent in comments about crime as something that exists everywhere—that the respondent’s neighborhood was not unique in that sense. They used extreme case formulations such as “all,” “wherever,” and “everywhere” (Pomerantz, 1986) to emphasize that crime was not unique to their neighborhood. Birgitta (69, Springfield) suggested there will always be problems wherever you live, and that “I don’t think there’s anywhere in Sweden better than the others.” She added that she regarded her neighborhood as a good place to live, but acknowledged that many would never consider moving there.

The respondents’ relativizing rhetoric usually followed on from the question of public reputation, and in several cases, they commented on how well they liked the area.

We like it here. What always happens is when we talk to people who call us, mostly right after that, “Have you heard there was a shooting in Springfield last night?” “No, we hadn’t heard” is what we say. We say it automatically to tease them a bit. Springfield is a big city today. It’s one of the 10 biggest in Sweden, and we can’t avoid the new lifestyle that comes with it … And that kinda makes people who live outside the cities, they react far more than we do who live here. We’re not worried (Stig, 79, Springfield).

Marta (78, Springfield) elaborated on the same tension, saying it was important not to say bad things about the neighborhood, but to tell outsiders that “we’re thriving in our area, and we have… because what is very … we have fantastic green spaces, and we have … and then I stress the good things.” Like several other respondents, she was careful to balance problematic images by mentioning the positive sides of the neighborhood.

## Discussion

The present study of two Swedish cities was based on interviews with 22 older adults and shows that crime and disorder directly influence residents’ daily routines, sense of security, and satisfaction with their neighborhoods. Some residents felt disappointed and dissatisfied, which left them less engaged in their neighborhood and longing to exit. Two third of the persons that were interviewed lived alone, making them particularly vulnerable, with a higher risk of poverty and isolation.

However, when applying an EVL framework (Hirschman, 1970; Permentier et al., 2007) it became clear that a number of residents were actively involved in formal and informal attempts to uphold order and reduce crime.

A number of studies report decreased well-being, isolation, and social exclusion among older people who live in socially deprived areas (Annear et al., 2014; Beard et al., 2009; Buffel et al., 2013; Choi & Matz-Costa, 2018; Dahlberg, 2020; Danielewicz et al., 2017; Rosso et al., 2011; Scharf et al., 2005; Shepperd et al., 2022; Wu et al., 2015). Our study implies a need to nuance descriptions of older people living in deprived areas. A second implication concerns the possibility to build on the activities and resources of older people when improving the conditions of these areas.

The study aimed to identify approaches and strategies that could be developed to further strengthen older adults’ contribution to socially deprived neighborhoods. While small in size, our findings highlight aspects of being an older person that could facilitate such efforts: spatial and temporal dimensions of attachment and connectedness. These aspects should be acknowledged as a complement to descriptions that frame the situation of older people as isolated; and perceiving a loss of community in socially deprived areas (Buffel et al., 2013; Dahlberg, 2020; Victor et al., 2009).

The fact that some older people have lived in their neighborhoods for a long time, and spend most of their time there because they are retired, is significant. As our study shows, having established social relations to others facilitated voice strategies. This connectedness took the form of being known as a friendly person, knowing children/youth and their parents, and knowing (older) neighbors who could act as backup, for example, when confronting drug dealers.

Regarding temporality, the expected description of problems associated with a changing population—for example, an influx of immigrants—was mentioned by many interviewees. Alongside such comments, several praised the community, singling out the support this very population of new residents had brought into the neighborhood, and some reflected on their own prejudiced attitudes in the past. What was evident in our study was that ideas about how the neighborhoods had developed, and would continue to do so, were central for the choice of strategy among residents. In a study of deprived neighborhoods in the Netherlands, Van Land and Doff (2010) argue that exit strategies are born of distrust and a sense of neglect. While most respondents in our study did not share that pessimistic view, the overlap between neglect and voice strategies seems relevant. Several expressed loyalty to their neighborhood and defended its reputation against outsiders (and the interviewers’ insinuations). In their explanations, acceptance and appreciation of the good things that characterized the neighborhood was set against prejudice, ignorance, and narrow-mindedness. Attachment, a social network among neighbors, access to services by living in a downtown area, and improvements by the municipality were all positives which kept respondents satisfied and facilitated their acceptance of the neighborhood’s daily challenges.

Feeling attached to the neighborhood is associated with the intention to remain and to use one’s voice when problems arise (Permentier et al., 2007; Van Land & Doff, 2010). Our findings show that neighborhood attachment among older people goes beyond the evaluation of a particular area. When describing their situation in the neighborhoods, the residents we interviewed commented on previous workplaces outside the area, adult children living in the neighborhood or elsewhere, membership of organizations, and their own previous places of residence. What they displayed was a broad context of past and present experiences and relations outside the area, that they still felt connected to. Arguably, older adults have a particular ability to hold balanced views on loyalty given their greater life experience, social inclusion, and contacts outside the area, and unlike the young people living in the same deprived neighborhoods their identities seem to include a broader “we,” not built on the antagonistic relations that characterize the typology proposed by Wacquant et al. (2014). This type of attachment beyond the neighborhood was also reflected in comments on improvements. The backup that some respondents arranged to feel safe when trying to uphold law and order was in a more generalized way described in their appreciation for how the police, the municipality, and the housing companies cared for the neighborhoods. In Springfield, respondents were largely positive when they contrasted images of deterioration with the local artworks or green spaces that gave them a sense of attachment, while several residents in Franklin felt insulted by the attempt to improve the reputation of the area by rezoning and replacing all services including the local shopping center by housing. Thus, it seems to indicate that older adults’ neighborhood attachment is linked to their perception of officialdom’s actions.

This is perhaps the most important lesson in relation to deprived neighborhoods and the way territorial stigma is played out. In Sweden, deprived neighborhoods have not been abandoned and allowed to deteriorate, unlike some other countries. Older adults who feel socially integrated in a society where efforts are made to improve conditions in their neighborhood may apply different voice strategies, designed to prevent crime and disorder and improve the reputation of the neighborhood.

### Methodological Limitations

Recruitment for the study was affected by the COVID-19 pandemic. In Springfield, respondents were recruited via an older adult center, the public library, and nonprofit organizations that promote integration and community. This method could not be used in Franklin; instead, addresses for all adults over the age of 65 were ordered from SPAR (the Swedish state personal address register), and a set of randomly chosen residents were sent a letter with information about the study, followed up with a phone call. This difference in sampling may have affected the difference in approach among residents of the two cities and, in particular, the profile of the group of socially engaged older adults in Springfield.

## Conclusion

There are socially deprived neighborhoods in many cities, and they are inhabited by individuals of all ages. Older adults are often stereotyped as vulnerable. Our findings establish the nuances in older peoples’ agency and how they choose to cope with everyday life, affecting territorial stigmatization, crime, and disorder. The findings show it is time to move away from generalized notions of older adults as passive victims of their environment to embrace the principle that they can be active agents in resistance to territorial stigmatization and in building communities in deprived neighborhoods. This has an important implication for policy decision-making. The engagement shown by older adults was not part of any traditional form of social movement, nor official city improvement program; nevertheless it exists, and we suggest it should be capitalized on by supporting older adults who wish to stay and engage in their neighborhoods by facilitating neighbor integration in local organizations. We also suggest supporting the older adults’ proactive role in improving their neighborhoods in joint efforts, as partners working together with municipalities, the police, and civil society organizations.

## Supplementary Material

gnac159_suppl_Supplementary_Material
