# Supplementary material for: Crime, Disorder, and Territorial Stigmatization: Older Adults Living in Deprived Neighborhoods
Source: Gerontologist. 2022 Oct 19;63(5):910–9. doi: 10.1093/geront/gnac159 (PMC10268582; doi:10.1093/geront/gnac159)
Supplement: gnac159_suppl_Supplementary_Material [file gnac159_suppl_supplementary_material.docx]

Section 1: Supplementary Table 1. The data analysis process (adapted from Braun & Clarke, 2012)

| Phases | Description of phases and the analytic process |
| --- | --- |
| 1. Familiarization with the data | All co-authors conducted interviews and contributed to the analysis. We inductively familiarized ourselves with the data during and immediately after the data collection phase. The subject of crime and disorder emerged—related to the residents’ everyday lives, attempts to uphold order, and reasoning about the neighborhood’s poor reputation—and we decided to concentrate on this specific subject. |
| 2. Generating initial codes | The phrases with bearing on the subject were extracted and coded by the first author using NVIVO software. The initial codes and the coding process were repeatedly discussed among the authors in analytic meetings.  Example of quote and initial codes:  “I know they’ve totally got my back, because when I’ve gone out there and… When the youngsters are hanging out at the corner of the other building, when I saw… when I go out to talk to them, I know that Kalle and Peggy are out on their balcony and Bill is out on his, watching, and then there are others watching, so they know… Yes, the youngsters know I have backup. We see you and we know who you are! So don’t do any of that shit!”  Initial codes: Not afraid to intervene, having back-up, youngsters cause trouble |
| 3. Searching for themes  Adding an analytic tool: the EVL framework | During analytic meetings among co-authors, codes were grouped into tentative themes such as criminal acts, maintaining safety, adapting everyday life, neighborhood’s poor reputation, and participants’ contributions and strategies.  The tentative themes showed, at first, puzzling contrasts in the data. Participants described horrible events and dangerous acts that they had encountered or witnessed. The responses, experiences, and strategies to those events were drastically different among the participants. Some participants expressed how the events had nothing to do with them and they appeared surprisingly distanced. Others surprised us by describing how they interfered in drug dealing attempts without another thought. To better understand these contrasting quotes, and complex patterns of strategies we implemented the EVL framework (Hirschman, 1970; Permentier, 2007) as an analytical tool.  Responses, experiences, and strategies to criminal events and disorder were sorted into exit, voice, or loyalty strategies. We then compared how loyalty to the neighborhood influenced both exit and voice strategies. We found exit strategies to be used both as means to continue daily life as well as they appeared as outcomes of people detaching themselves from the neighborhood.  As a result, four themes were developed:  crime and disorder as a part of everyday life  exit, adaptations, and detachment  voice strategies  the neighborhood’s poor reputation – distancing and loyalty |
| 4. Reviewing potential themes | All co-authors discussed and reviewed the developed themes in relation to the tentative themes, coded data, and transcripts. For example, we thoroughly discussed how concepts like exit, neglect and detachment played out in the data and how loyalty was related to contrasting responses, experiences, and strategies. |
| 5. Defining and naming themes | We decided on definitions of the themes, which quotes to use to present them, and finalized naming the themes. At this stage, the quotes in Swedish and in Persian were translated into English. The translations were agreed upon co-authors, underwent professional language editing, and then reviewed again by the co-authors. |

*Note*. EVL = Exit, Voice, Loyalty framework .

Section 2: Interview Questions

1. Please, let us start with you telling me about yourself and what it is like to live in this neighborhood?

2. When I say “neighborhood”, how do you define it? What is your neighborhood? Is it [name of the deprived area]? Or is your neighborhood only a part of that? Who do you consider your neighbors?

3. Why do you live here? Would you like to share with me, the story behind your decision to move here?

4. The police labeled your neighborhood a deprived neighborhood. It is in the news from time to time too. Did you know of it? Have you seen such news? What do you think of it? Do you agree? Is it fair? What do you think when you read about your local area in the newspaper?

5. You said you had lived here for [xx] years. Have you changed since you moved here? (Health, growing older, changed roles, changed habits?)

6. What do you think about your health status? Do you think your neighborhood influences your health or ability to do things and to socially participate?

7. Let´s turn to your apartment. How do you feel about your apartment? Do you feel at home here - or where would you feel at home? Is it practical/functional for you?

8. How do you think people who do not live, think and talk about your neighborhood? What do you think about it?

9. How would you describe the atmosphere in the neighborhood? Do you consider your neighborhood being a community? How so? Are you a part of it? In what ways? How are neighbors treating each other? Do you think of your neighbors as your friends?

10. What does this place mean to you? Throughout life, have there been other places that were meaningful to you? Can you rate them in relation to each other? Has it changed (over time)?

11. Do you think that your neighborhood has changed since you moved here and if so, how? Are there things that have not changed?

12. Have the changes you mentioned influenced your daily activities outside the home in any way? Your level of independence? Social participation?

13. Are there activities that you wish you could do, or places you want to visit in the neighborhood that you cannot due to the situation in the area? What would you miss the most if you moved away?

14. Is there something you would like to change in this area?

15. Are you involved in any community groups, civic engagement? How do you think each resident living here can contribute a sense of community?

16. How do you see the years ahead? Will you stay here? Move? Where do you see yourself in 5 years?

17. What do you think about the future of the neighborhood? Where do you see the neighborhood in 5 years?
